# Strategies for Rapid Identification of *Acinetobacter baumannii* Membrane Proteins and Polymyxin B’s Effects

**DOI:** 10.3389/fcimb.2021.734578

**Published:** 2021-09-21

**Authors:** Yun Lu, Xinxin Hu, Tongying Nie, Xinyi Yang, Congran Li, Xuefu You

**Affiliations:** Beijing Key Laboratory of Antimicrobial Agents, Institute of Medicinal Biotechnology, Chinese Academy of Medical Sciences and Peking Union Medical College, Beijing, China

**Keywords:** *Acinetobacter baumannii*, membrane proteome, polymyxin B, detergents, LC-MS/MS

## Abstract

*Acinetobacter baumannii*, especially multidrug resistant *Acinetobacter baumannii*, is a notable source of pressure in the areas of public health and antibiotic development. To overcome this problem, attention has been focused on membrane proteins. Different digestion methods and extraction detergents were examined for membrane proteome sample preparation, and label-free quantitative and targeted proteome analyses of the polymyxin B-induced *Acinetobacter baumannii* ATCC 19606 membrane proteome were performed based on nano LC-MS/MS. Ultracentrifugation of proteins at a speed of 150,000×*g*, digestion by trypsin, filter-aided sample preparation, and detergents such as lauryldimethylamine-N-oxide were proved as a fast and effective way for identification of membrane proteome by nano LC-MS/MS. Upon treatment with polymyxin B, expression levels of 15 proteins related to membrane structure, transporters, cell surface, and periplasmic space were found to be significantly changed. Furthermore, targeted proteome was also used to confirm these changes. A relatively rapid membrane proteome preparation method was developed, and a more comprehensive view of changes in the *Acinetobacter baumannii* membrane proteome under polymyxin B pressure was obtained.

## Introduction

*Acinetobacter baumannii* (*A. baumannii*) is a Gram-negative bacterium that can cause serious nosocomial infection (Lin and Lan, 2014) and has developed extensive antimicrobial resistance with a high mortality rate (Vazquez-Lopez et al., 2020). The World Health Organization (WHO) reported that *A. baumannii* was one of the most serious ESKAPE organisms (*Enterococcus faecium*, *Staphylococcus aureus*, *Klebsiella pneumoniae*, *A. baumannii*, *Pseudomonas aeruginosa*, and *Enterobacter cloacae*) which could effectively escape the actions of antibacterial drugs (Boucher et al., 2009). With the increase in multidrug resistance (MDR), *A. baumannii* showed resistance to many first-line antibiotics, even colistin and polymyxin B (Olaitan et al., 2014).

In bacteria, approximately 20% to 30% of all genes encode membrane proteins. Membrane proteins play vital roles not only in defense and immunity but also in essential physiological functions, such as the generation or conversion of energy, the import or export of metabolites, the extrusion of toxic substances or antibiotics, and the homeostasis of metal ions (Ansgar and Dirk, 2010). Many membrane proteins were found to be related to drug resistance or potential therapeutic targets, such as OmpA in *A. baumannii* (Bunpa et al., 2020).

In the past few decades, attention has been given to the bacterial membrane proteins, and proteome techniques for the detection of membrane proteins have been developed to help to identify the classes of proteins. Previous studies reported that the use of SDS-PAGE and LC-MS/MS to separate and detect bacterial membrane proteins (Ghosh et al., 2008) and 135 membrane proteins and 23 periplasmic proteins in *A. baumannii* ATCC 19606 were identified (De et al., 2011). Then two-dimensional gel electrophoresis (2-DE) was used to improve the separation efficiency, and 29 major protein spots were selected for MS analysis (Cordwell, 2006; Marti et al., 2006). In recent years, with the improvement of mass spectrometry, shotgun proteomics based on peptide detection combined with quantitative methods has been developed and widely used in protein identification and the comparison of protein expression levels (Boumediene and Boris, 2015). Currently, the quality and quantity of protein identification are further improved with the optimization of sample preparation methods such as filter-aided sample preparation (FASP), and the emergence of high-resolution mass spectrometers.

However, apart from the promotion of equipment and methods for detection, efficient and effective membrane protein extraction methods are essential for membrane peptide/protein identification. Characterization of the membrane proteome is challenging due to the hydrophobic nature of membrane proteins. Techniques such as density gradient centrifugation and ultracentrifugation have also been developed to separate membrane complexes (Papanastasiou et al., 2016). Amphiphilic molecules, such as detergents, are the main reagents used to extract membrane proteins, which can replace and imitate the stabilizing properties of natural phospholipids to avoid irreversibly disrupting the protein structure (Gao et al., 2017). According to the characteristics of their polar groups, detergents are generally divided into ionic, nonionic, and zwitterionic categories (Liu et al., 2015). Various studies have evaluated the effectiveness of extraction for targeted membrane proteins (Arachea et al., 2012). However, there are no guidelines that can recommend which detergent is the most effective for extracting a given protein class or total proteome from the membrane. Therefore, although the use of detergent-based proteomics analysis has produced a large amount of useful data, there is still an urgent need for novel methods that are effective for the overall membrane proteome and are not affected by detergent side effects. In our study, we evaluated the effectiveness of different digestion enzymes, digestion conditions, and detergents [ionic (sodium deoxycholate, SDC), nonionic (dodecyl-b-D-maltoside, DDM, or n-octyl-b-D-glucoside, OG), and zwitterionic detergents (lauryldimethylamine-N-oxide, LDAO or 3-[(3-cholamidopropyl)dimethylammonio]-1-propanesulfonate, CHAPS)] in *A. baumannii* membrane proteome analysis by nano LC-high resolution MS and developed an effective and rapid workflow for *A. baumannii* membrane proteome analysis.

Polymyxins have been treated as a last choice for the therapy of serious infections caused by multidrug-resistant Gram-negative bacteria, particularly *A. baumannii* (Nang et al., 2021). Many studies have focused on the mechanism of polymyxins in bacteria, and some have elucidated the molecular and chemical interactions between polymyxins and lipopolysaccharide structure (Ayoub Moubareck, 2020). However, there is little research observing the effect of polymyxin B on *A. baumannii* from the perspective of the whole membrane proteome by high-resolution MS. Thus, in our study, label-free quantitative proteome and parallel reaction monitoring (PRM) targeted proteome were used to explore the differentially expressed membrane proteins in polymyxin B treated *A. baumannii* ATCC 19606.

## Material and Methods

### Bacterial Strains and Materials

*Acinetobacter baumannii* (*A. baumannii*) ATCC19606 was purchased from American Type Culture Collection (ATCC) (Manassas, USA). OG, LDAO, DDM, CHAPS, and SDC were purchased from Sigma-Aldrich (St. Louis, MO). Trypsin Gold, Mass Spectrometry Grade, and Trypsin/Lys-C Mix, Mass Spec Grade, were purchased from Promega (WI, USA). Nanosep^®^ Centrifugal Devices with Omega™ Membrane 10K were purchased from Pall Nanosep (Ann Arbor, MI).

### Cell Culture and Sample Preparation

*A. baumannii* ATCC19606 was cultivated in Luria–Bertani (LB) broth at 37°C with shaking (220 rpm) until OD600 reached 0.6. Bacterial cells were harvested by centrifugation at 4,000×*g* for 10 min and washed with 0.85% NaCl. Bacterial precipitation was frozen at -80°C until use. *A. baumannii* cells were resuspended in Na_2_CO_3_ buffer (0.1 M) for sonication at 4°C. Cell debris was removed by centrifugation (12,000×*g* for 10 min, 4°C). Supernatants were transformed into thick-walled tube and ultracentrifuged at 50,000 or 150,000×*g* for 30 min. The previous steps were repeated twice to wash off non-membrane proteins as much as possible. Then, the detergents (4% CHAPS, 1% DDM, 2% SDC, 0.5% OG, and 1% LDAO) were added and incubated at 4°C overnight, after which ultracentrifugation was performed at 50,000×*g* for 30 min. Suspensions were collected for peptide preparation. After reduction by 10 mM dithiothreitol (DTT) at 95°C for 5 min, proteins were precipitated by frozen acetone and stored at -20°C for 30 min. Then, the precipitate was redissolved by 50 mM ammonium bicarbonate, then digested in solution by sequencing grade trypsin (1:50 w/w, Promega, Madison, WI) or trypsin/Lys-C Mix (1:25 w/w, Promega, Madison, WI) overnight at 37°C. For the FASP method, precipitate redissolved by 50 mM ammonium bicarbonate were transfer on ultrafiltration tube, centrifuged at 14,000×*g* for 30 min. Digestions were performed by microwaving for 2 min and then overnight at 37°C in 50 µl 50 mM ammonium bicarbonate with trypsin or trypsin/Lys-C Mix. Last, the sample was desalted by C18 reverse-phase Tips (ReproSil-Pur Basic C18, 5 µm, Dr. Maisch GmbH). For the study on *Acinetobacter baumannii* treated with polymyxin B, *A. baumannii* ATCC19606 was cultivated until OD600 reached 0.5, then polymyxin B was added (0.5 µg·ml^-1^) and bacteria were cultivated at 37°C for 1.5 h. Four biological replicates were performed, and both control and treatment groups were collected and lysed. Ultracentrifugation of proteins at a speed of 150,000×*g*, digestion by trypsin, filter-aided sample preparation, and lauryldimethylamine-N-oxide were used for membrane peptide acquisition.

### Nano LC−MS/MS and Data Acquired

Samples were analyzed on a Thermo Scientific Orbitrap Fusion Lumos platform. The trap and separation columns along with the composition of solvent buffer A and B used in nano LC were the same as previously reported (Lu et al., 2021). Furthermore, a 75-min elution method was set as follows; the gradient started from 10% of solvent buffer B (80% acetonitrile with 0.1% formic acid) and then from 10% to 13% of solvent buffer B for 4 min. The gradient rose from 13% to 29% of solvent buffer B for 48 min and from 29% to 37% for 14 min. Ultimately, the gradient ascended to 100% of buffer B and was maintained for 9 min. The scan parameters of MS1 and MS2 were set as previously reported (Lu et al., 2021) with high energy collision-induced dissociation (HCD) as the activation type and Orbitrap as the detector. Data-dependent acquired data with four biological replicates were collected and searched against the *Acinetobacter baumannii* ATCC 19606 database downloaded from UniProt. The search parameters were set as previously reported (Lu et al., 2021), and the FDR was limited to a maximum of 0.01. Protein identification and label-free quantification (LFQ) analysis were all analyzed by Thermo Scientific™ Proteome Discoverer™ version 2.2 (PD2.2). The nano LC-MS/MS proteomics data have been deposited to the ProteomeXchange Consortium *via* the PRIDE (Perez-Riverol et al., 2019) partner repository with the dataset identifier PXD026714.

### Bioinformatics Analysis

The counts of protein groups, peptide groups, PSMs, and MS/MS spectra were analyzed by PD 2.2, comparison between groups were performed by t test or one-way ANOVA. Venn analysis was used to compare the efficiency of different enzyme digestion methods to identify proteins. GRAVY and amino acid components were used to evaluate the peptides identified by different detergents used in extraction (Gao et al., 2017). For polymyxin B-induced *A. baumannii* ATCC19606, label-free quantitative analysis was performed as previously reported (Lu et al., 2021). In brief, missing value processing, CV limitation, and hypothetical tests were applied and proteins with fold change >1.5 and p < 0.05 were considered significantly changed. Differentially expressed proteins were annotated and clustered for enrichment by the Gene Ontology enrichment tool. In view of the lack of the *A. baumannii* ATCC19606 KEGG database, the database of *A. baumannii* AYE was used as both the GO annotation and KEGG pathway databases were relatively complete. The KEGG pathway database of *A. baumannii* AYE was used to aid in discovering the affected pathways under polymyxin B pressure.

### Targeted Proteome Analysis

PRM, a targeted proteome method that can be performed by the nano LC-Orbitrap Fusion Lumos platform, was employed to verify the differentially expressed proteins. The extraction and preparation methods were the same with the label-free proteome analysis, and four biological replicates were generated. More than two peptides were selected for each protein, and peptides with missed cleavage sites and amino acid residues susceptible to modification such as M were excluded. Parameters of peptides (retention time, precursor m/z, and charge state) were exported by Skyline software according to the previous data-dependent acquisition (DDA) experiments. Moreover, the nano LC parameters were the same with that previously described. PRM was monitored by an Orbitrap detector, and a 0.7-m/z isolation window was used, as previously reported (Lu et al., 2021). PRM results were analyzed by Skyline with DDA results served as spectral library. Peptides were selected by t test with p-value < 0.05 and fold change >1.5.

## Results

### Effects of Ultracentrifugation and Digestion Enzymes on Membrane Protein Identification

**Figure 1:**
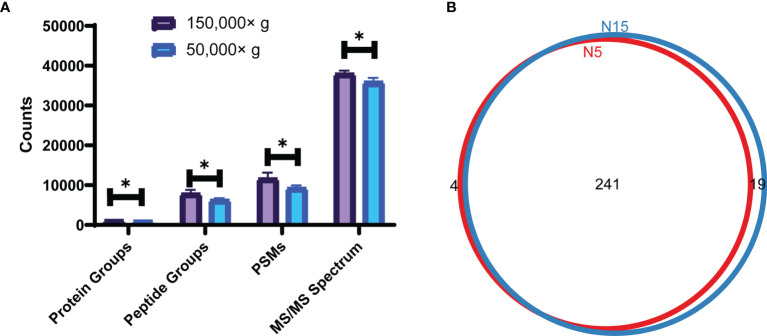
Analysis results for ultracentrifugation at the speed of 150,000×g and 50,000×g **(A)** The counts of total protein groups, peptide groups, PSM, and MS/MS spectrum at speeds of 150,000×g and 50,000×g; comparison between groups was performed by t test, (*) p < 0.05. **(B)** Venn plot of membrane proteins identified at speeds of 150,000×g(N15) and 50,000×g(N5).

**Figure 2:**
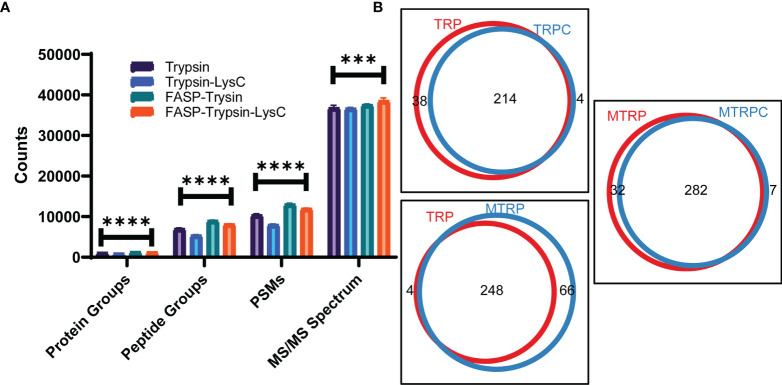
Analysis results for different digestion methods. **(A)** Counts of total protein groups, peptide groups, PSM, and MS/MS spectrum of trypsin and trypsin-LysC digestion in solution or by FASP; comparison between groups was performed by one-way ANOVA, (***) p < 0.001, (****) p < 0.0001. **(B)** Venn plot of membrane proteins identified of trypsin (TRP) and trypsin-LysC digestion (TRPC) in solution or trypsin (MTRP) and trypsin-LysC digestion (MTRPC) by FASP.

As shown in **Figure 1**, the number of protein groups, peptide groups, PSM, and MS/MS spectra at the speed of 150,000×*g* were higher than those obtained at 50,000×*g*. For membrane protein identification, there were 241 proteins common across the two groups, and 4 exclusive to the 50,000×*g* group and 19 exclusive to the 150,000×*g* group (**Supplementary Table 1**). Digestion in solution by trypsin was less effective than digestion on the membrane by trypsin with fewer protein groups, peptide groups, peptide spectrum match (PSM), and MS/MS spectrum (**Figure 2A**). Interestingly, the addition of LysC did not improve the digestion quality and quantity. Then, membrane proteins identified in different groups were compared to obtain the counts of overlap (**Figure 2B** and **Supplementary Tables 2–4**).

### Detergent Efficiencies of Membrane Protein and Peptide Identification

**Figure 3:**
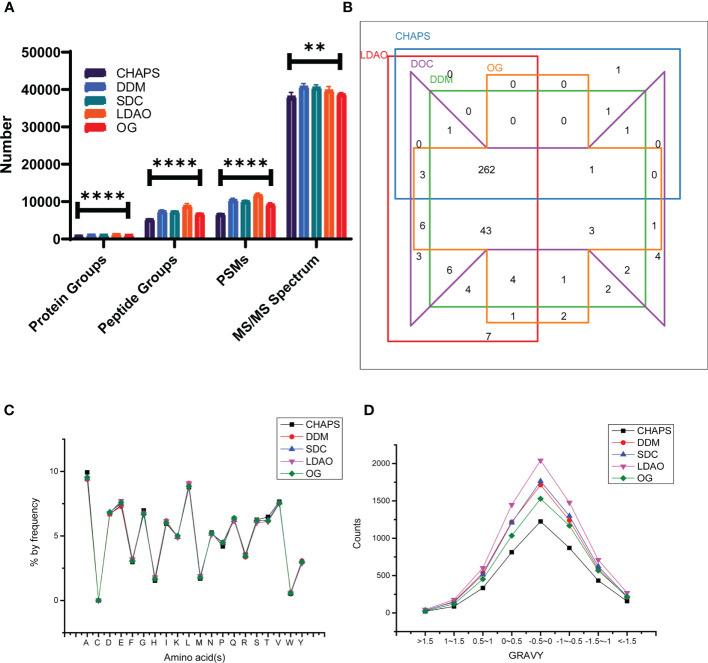
The performances of CHAPS, DDM, SDC, LDAO, and OG for membrane protein lysis and extraction. **(A)** The identified counts of total protein groups, peptide groups, PSM, and MS/MS spectrum using different detergents; comparison between groups was performed by one-way ANOVA, (**) p < 0.01, (****) p < 0.0001. **(B)** Venn plot of membrane proteins identified using different detergents. **(C)** Amino acid distribution of peptides identified using different detergents. **(D)** GRAVY values of peptides identified using different detergents.

We investigated the performances of different detergents including CHAPS, DDM, SDC, LDAO, and OG for membrane protein lysis and extraction. First, as shown in **Figure 3A**, the number of protein groups, peptide groups, and PSM of the LDAO group were highest among the five groups, and the number of MS/MS spectra in the DDM group was highest. Then, the membrane proteins identified in different groups were compared to acquire the numbers of overlaps (**Figure 3B**), 339 membrane proteins were identified in LDAO group, while 269, 330, 335, and 326 membrane proteins were identified in CHAPS, DDM, DOC, and OG groups, respectively (**Supplementary Table 5**). We further compared the amino acid distribution and grand average of hydropathy (GRAVY) scores, and the curves of amino acid distribution curves were similar among groups (**Figure 3C**). Hydrophobic peptides (peptides with positive GRAVY values) and hydrophilic peptides (peptides with negative GRAVY values) were identified and classified, as shown in **Figure 3D**. All groups showed a higher proportion of hydrophilic peptides. Interestingly, the curves of the GRAVY score for DDM and SDC were similar, while that of LDAO was the highest.

### Comparative Membrane Proteomics Under Polymyxin B Pressure

**Figure 4:**
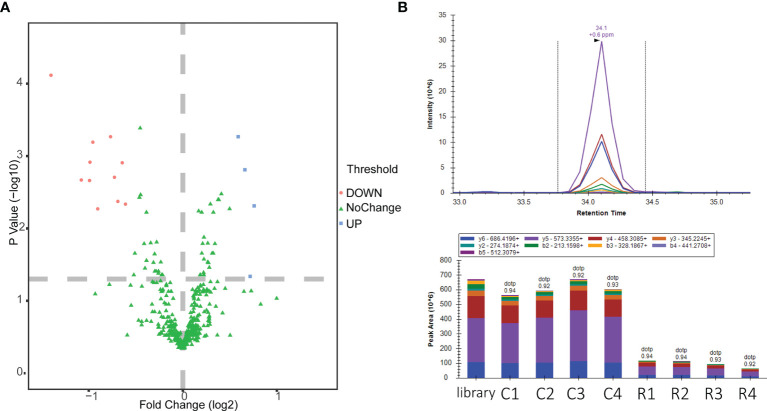
Label-free quantitative analysis of polymyxin B-induced *(A) baumannii* ATCC19606. **(A)** Volcano plot of scaled values for proteins. Red indicates upregulation, and green indicates downregulation. **(B)** Chromatogram of PRM fragment ions for VLDLAVR and the peak area comparison of polymyxin B-treated and untreated groups. Control groups: C1, C2, C3, and C4; polymyxin B-treated groups: R1, R2, R3, and R4.

Optimized membrane protein extraction and peptide preparation methods were applied to the proteome analysis of polymyxin B-induced *A. baumannii* ATCC 19606. A previous study has found that polymyxin B inhibited *A. baumannii* ATCC 19606 at a minimum inhibitory concentration (MIC) of 1 μg·ml^-1^ (Cui et al., 2020). In our study, a sublethal concentration of polymyxin B (0.5 μg·ml^-1^) was used and four biological replicates were performed. In total, 2,000 proteins were identified with a cutoff of FDR <0.01 (**Supplementary Table 6**). Proteins with a CV value greater than 30% and a large proportion of missing values were excluded, and 421 membrane proteins (**Supplementary Table 7**) including membrane structure proteins and proteins located in the cell surface or periplasmic space and transporter were identified in both groups. Good repeatability was verified by linear correlations with R^2^ > 0.9 (**Supplementary Figure 1**). The expression levels of 15 membrane proteins were identified as significantly changed under polymyxin B pressure (**Figure 4A**) based on a max fold change > 1.5 and adjusted p value (p < 0.05). The expression levels of 11 membrane-related proteins reduced while 4 proteins increased under polymyxin B pressure.

All 15 significantly changed membrane proteins (**Supplementary Table 8**) were analyzed by gene ontology (GO) annotation enrichment and Kyoto Encyclopedia of Genes and Genomes (KEGG) pathway enrichment. As shown in **Supplementary Figure 2**, most of the enriched proteins were related to transport, localization, cellular anatomical entity, membrane, binding, etc. Due to the lack of the KEGG pathway database of *A. baumannii* ATCC19606, we aligned all the proteins to *A. baumannii* strain AYE to perform the KEGG pathway enrichment. According to the analysis results, D0CAD3 and D0C805 were involved in the ABC transporter pathway while D0CBP8 and D0C805 were involved in the two-component system pathway, indicating the extensive effects of polymyxin B on the membrane proteins of *A. baumannii* ATCC19606. We further quantified 5 proteins with 15 peptides (**Supplementary Table 9**, **Figure 4B**) as significantly differentially expressed proteins (p value < 0.05 and fold change >1.5) by PRM targeted proteomics.

### Discussion

Recently, given the confirmation of the roles of cell membrane proteins in bacterial survival, defense, immunity, and drug resistance, interest has been focused on bacterial cell membrane proteins (Ansgar and Dirk, 2010). However, the complexity and specificity of membrane protein structures limit the extraction efficiency, and effective methods need to be developed. Moreover, the low abundance of membrane proteins and separation methods such as 1-DE or 2-DE has led to a limited number of identified proteins (Cordwell, 2006). As quantitative proteomics based on LC-high resolution MS has been widely used in studies related to pathogenic microorganism, compatible bacterial membrane extraction and preparation methods need to be developed.

In our study, ultracentrifugation combined with Na_2_CO_3_ was used to separate membrane proteins (Zhang et al., 2008). The extraction efficiency of proteins and peptides at a speed of 150,000×*g* was much higher than that at a speed of 50,000×*g*. Different protein digestion methods were also verified, and the digestion efficiency of by the FASP method was much higher than that of the in-solution digestion. Moreover, although studies have determined that LysC could help improve the digestion efficiency of the total proteins (Hakobyan et al., 2019), for membrane proteins, LysC decreased the digestion efficiency of both the FASP and in-solution digestion methods. We further classified the proteins by GO annotation enrichment. A total of 245 and 260 membrane proteins were obtained separately at speeds of 50,000×*g* and 150,000×*g*, respectively. A total of 252, 218, 314, and 289 membrane proteins were acquired separately for the trypsin (in solution), trypsin-LysC (in solution), trypsin-FASP, and trypsin-LysC-FASP methods, respectively.

Detergents are commonly used in membrane protein extraction. DDM is a mild detergent with the ability to purify membrane proteins and solubilize hydrophobic proteins without changing their structures (Liu et al., 2015; Lu et al., 2021). SDC, which is friendly to trypsin and can be removed easily from the peptides solution, demonstrated good ability in membrane protein extraction (Moore et al., 2016). CHAPS, LDAO, and OG were commonly used in proteome extraction (Arachea et al., 2012). In our study, we evaluated the extraction efficiencies of CHAPS, DDM, SDC, LDAO, and OG on the *A. baumannii* membrane proteome. To be consistent, all detergents were removed by acetone precipitation. As shown in **Figure 3A**, the extraction efficiencies of DDM, SDC, and LDAO were much higher than those of CHAPS and OG. Peptides identified in five groups were also analyzed. Amino acid distributions of the five groups were similar. For GRAVY values, as shown in **Figure 3D**, the peptides were mainly distributed in three sections: (−1.0, −0.5], (−0.5, 0], and (0, 0.5]. The range of the peptides identified indicated that both hydrophobicity and hydrophilicity could be identified by the five detergents. The counts of peptides obtained with LDAO were highest in all sections, which suggested that LDAO was the most efficient detergent. According to the GO enrichment results, 269, 330, 335, 339, and 326 membrane proteins were acquired separately for CHAPS, DDM, SDC, LDAO, and OG, indicating the different extraction abilities of the membrane proteins of the five detergents. Previous studies have reported that DDM is a detergent that has little influence on the enzyme activity and does not need to be removed from samples for proteomic analysis (Liu et al., 2015). Furthermore, SDC can precipitate in 1% formic acid (Lu et al., 2021), which can be easily separated from the peptide buffer. In view of the peptide loss that may be caused by detergent removal, SDC and DDM can also be considered as preferred detergents for the extraction of membrane proteins.

As the last-resort antibiotics for the treatment of MDR Gram-negative bacteria, polymyxin B has received notable attention. Previous studies have reported partial mechanisms of action of polymyxin B on Gram-negative bacteria, such as binding to LPS and oxidative bursts. Recently, outer membrane vesicles (OMVs) released from *A. baumannii* were reported as decoys against polymyxin B, whose production is linked to membrane-linkage proteins (Park et al., 2021). However, the mechanism of action of polymyxin B in *A. baumannii*, which can live without LPS, still needs to be explored. In our research, the membrane proteome changes of polymyxin B-induced *A. baumannii* ATCC 19606 were studied based on nano LC combined with high-resolution MS. Compared to the control group, 15 significantly differentially expressed proteins were acquired and clustered by bioinformatics methods. Among the 15 membrane proteins, the reduced expression of SurA (D0C7T2), which is important for the biogenesis of β-barrel outer membrane proteins (OMPs), would damage the homeostasis and functions of the cell envelope (Behrens-Kneip, 2010; Humes et al., 2019). The expression level of OmpA family protein (D0C8N6), a major component of OMPs, is suppressed, which interrupts bacterial antibiotic resistance, infection, and immunomodulation (Bunpa et al., 2020). What is more, OmpA was reported to be identified in the colistin-resistant *Enterobacter asburiae* (Ayoub Moubareck, 2020), indicating the potential important roles of OmpA in the mechanism of actions and resistance development. The reduced expression level of glutamate/aspartate transport system permease protein GltK(D0C805) will affect the glutamate/aspartate uptake and metabolism and bile resistance (Zhang et al., 2013). The reduced expression level of CsgG (D0CE32) lowers the curli biogenesis and affects the biofilm formation (Zhang et al., 2020). The suppressed expression level of the TonB-dependent siderophore receptor (D0CC21) affects iron ion homeostasis (Fujita et al., 2019). Moreover, the downregulation of putative ATP synthase F0 (D0C7A7), C4-dicarboxylate transporter (D0CF35), and carbonate dehydratase (D0CCK7) influences the membrane transport, energy consume and metabolism. The reduced expression levels of two proteins, which were located in periplasmic space (D0CBP8, periplasmic serine endoprotease DegP-like; D0CBL3, Tol-Pal system protein TolB) (**Supplementary Table 8**), indicated the destruction of the periplasmic space. Moreover, the reduced expression level of tolB affects the Tol-Pal system, secretion of EspA/B, and infection (Hirakawa et al., 2020). Interestingly, the expression levels of three proteins increased in the polymyxin B-treated group, including signal peptide peptidase SppA (D0CBY0), GTPase Era (D0CBQ4), and MacB (D0CAD3), part of the MacAB-TolC complex. A previous study suggested that R-LPS, which can be bound by polymyxin B, is a physiological substrate of MacAB-TolC (Lu and Zgurskaya, 2013). However, due to the imperfect KEGG pathway database (*A. baumannii* strain AYE), only D0CAD3 and D0C805 were involved in the ABC transporter pathway while D0CBP8 and D0C805 were involved in the two-component system pathway. The upstream and downstream genes of the pathway are currently not annotated in the *A. baumannii* ATCC 19606 genome, which hindered us from further verifying the pathway affected by polymyxin B.

In general, the membrane proteome based on nano LC-MS/MS is an effective tool for research related to membrane proteins. As mentioned above, we developed a relatively rapid membrane protein extraction and preparation method, but there were still many non-membrane proteins mixed in, partially because of the super-high identification ability of high-resolution MS. According to the label-free results, the abundance of membrane-related proteins is about 50% of the total abundance of each group. The purity can meet the needs of the proteome analysis at present, but better extraction methods should be explored. Moreover, our study found that polymyxin B could decrease the expression of many membrane proteins, including inner, outer, and trans-membrane proteins, which provided new evidence for the mechanism of actions of polymyxin B on the *A. baumannii* ATCC 19606 membrane. In turn, the discovery of targets will further promote the development of new antibiotics.

In summary, a relatively rapid membrane proteome sample preparation method of *A. baumannii* was built and applied for nano LC-MS/MS analysis. Through optimized quantitative membrane proteome analysis, 15 membrane proteins were found to be significantly changed under the pressure of polymyxin B, which gives us a better understanding of the role of polymyxin B and can help us further discover the mechanism of action of polymyxin B from the perspective of membrane proteins.

## Data Availability Statement

The data presented in the study are deposited in the PRIDE repository, accession number PXD026714. The names of the repository/repositories and accession number(s) can be found in the article/**Supplementary Material**.

## Author Contributions

Conceptualization, XFY and YL. Methodology, YL. Software, YL. Validation, YL and TN. Resources, XH. Data curation, YL. Writing—original draft preparation, YL. Writing—review and editing, XFY, XYY, and CL. Visualization, YL. Supervision, XH. Project administration, XFY. funding acquisition, XFY and YL. All authors contributed to the article and approved the submitted version.

## Funding

This research was funded by the National Natural Science Foundation of China (grant numbers 81803593, 81621064), the CAMS Initiative for Innovative Medicine (grant number 2016-I2M-3-014), the National Mega-project for Innovative Drugs (grant number 2019ZX09721001), the MOST/MOF Funds for the National Science and Technology Resource Sharing Service Platform, China (2019-194-NPRC), and the Fundamental Research Funds for the Central Universities (grant number 3332018094).

## Conflict of Interest

The authors declare that the research was conducted in the absence of any commercial or financial relationships that could be construed as a potential conflict of interest.

## Publisher’s Note

All claims expressed in this article are solely those of the authors and do not necessarily represent those of their affiliated organizations, or those of the publisher, the editors and the reviewers. Any product that may be evaluated in this article, or claim that may be made by its manufacturer, is not guaranteed or endorsed by the publisher.
