# Supplementary material for: Strategies for Rapid Identification of Acinetobacter baumannii Membrane Proteins and Polymyxin B’s Effects
Source: Front Cell Infect Microbiol. 2021 Sep 21;11:734578. doi: 10.3389/fcimb.2021.734578 (PMC8490878; doi:10.3389/fcimb.2021.734578)
Supplement: Supplementary file 1 [file DataSheet_1.docx]

Supplementary Material

Strategies for Rapid Identification of *Acinetobacter baumannii* Membrane Proteins and Polymyxin B’s effects

Yun Lu , Xinxin Hu , Tongying Nie , Xinyi Yang , Congran Li , and Xuefu You *

Beijing Key Laboratory of Antimicrobial Agents, Institute of Medicinal Biotechnology, Chinese Academy of Medical Sciences and Peking Union Medical College, Beijing 100050, China

* Correspondence: xuefuyou@imb.pumc.edu.cn (X.Y.); Tel.: +86-10-67061033 (X.Y.); Fax: +86-10-67017302 (X.Y.)

## Supplementary Figures


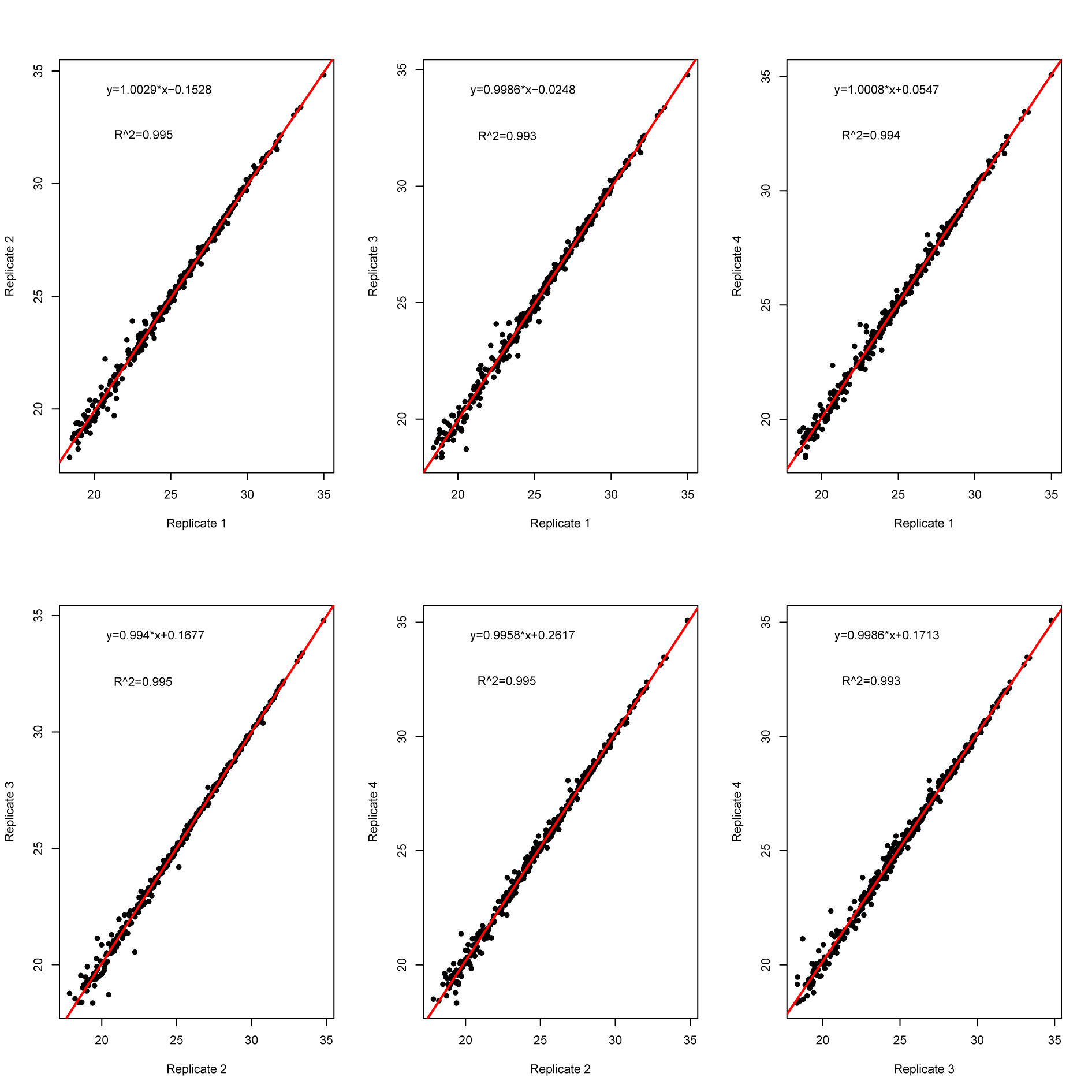


(a)


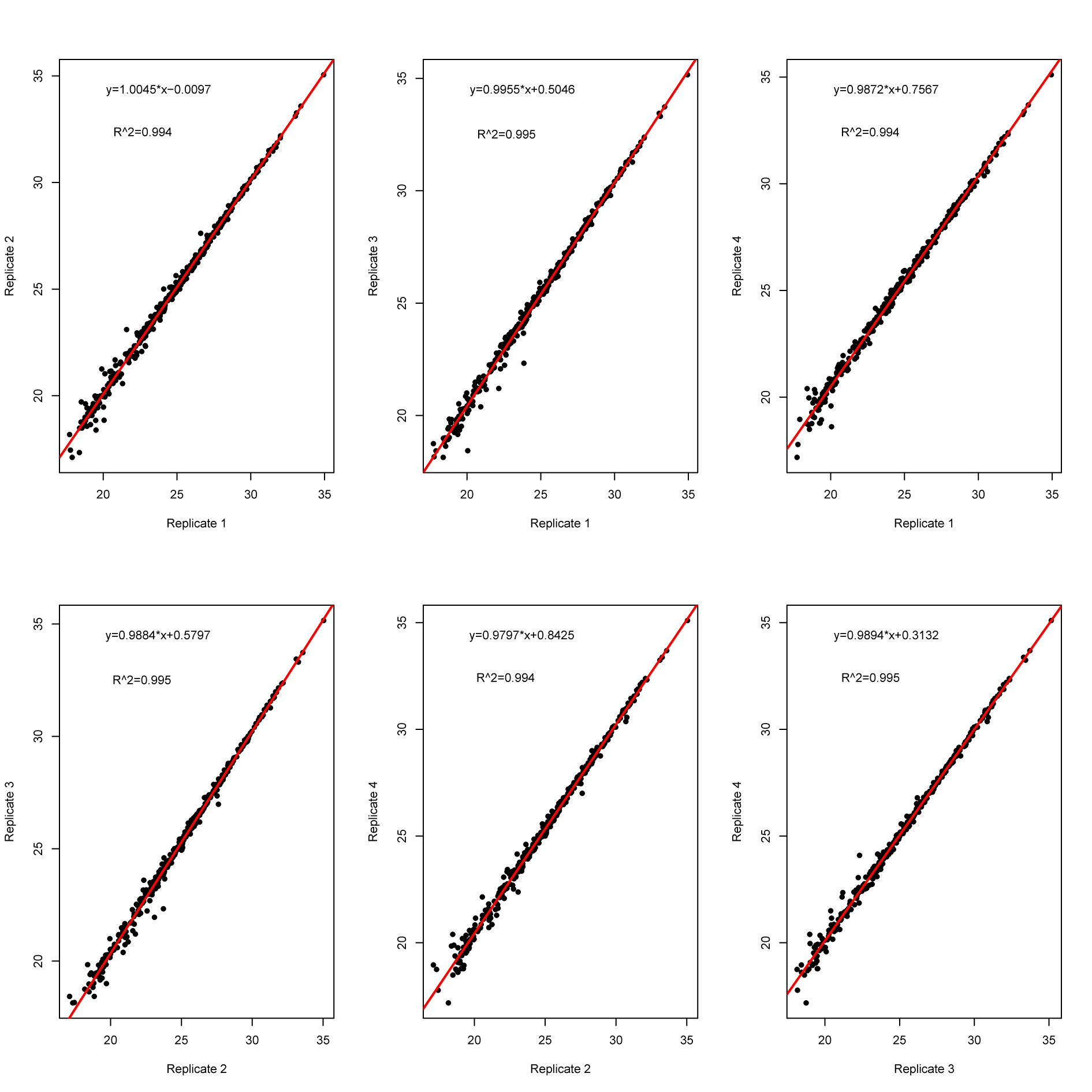


**Supplementary Figure 1.** Linear correlations curves of biological replicates of label-free quantitative analysis of *A.baumannii* ATCC19606 under polymyxin B pressure.


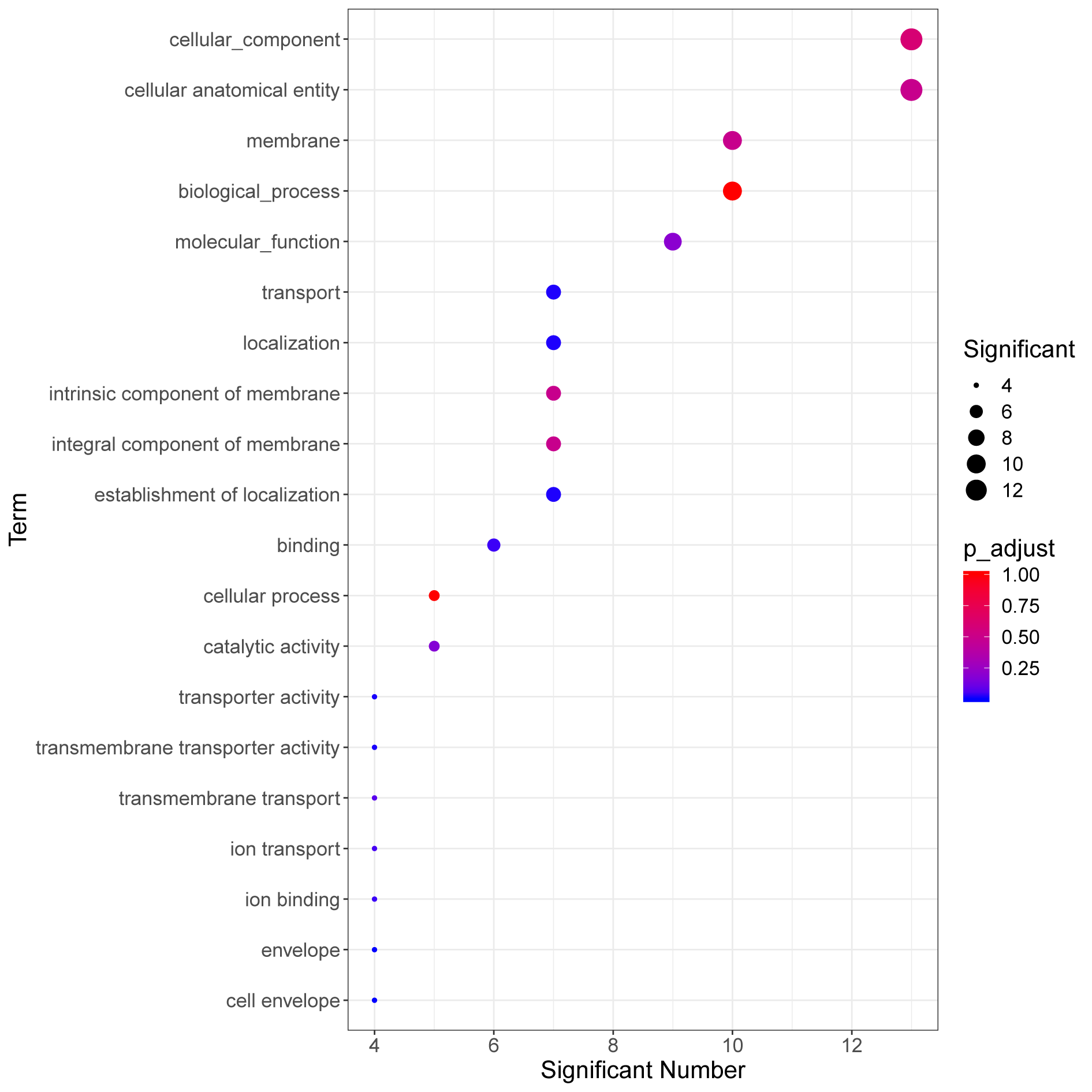


**Supplementary Figure 2.** GO enrichment results of differently expressed membrane proteins of *A.baumannii* ATCC19606 under polymyxin B pressure.

**Supplementary Tables.**

**Supplementary Table 8.** The list of significantly differentially expressed membrane-related proteins under polymyxin B pressure (filtered by p˂0.05, fold change˃1.5).

| **Uniprot ID**  **(***A.baumannii* ATCC19606**)** | **Protein name** | **Fold.Change** | **p value** |
| --- | --- | --- | --- |
| D0C7T2 | Chaperone SurA (Peptidyl-prolyl cis-trans isomerase SurA) (PPIase SurA) (EC 5.2.1.8) (Rotamase SurA) | 0.604224493 | 0.001964881 |
| D0CAD3 | Macrolide export ATP-binding/permease protein MacB (EC 7.6.2.-) | 1.503339584 | 0.000540661 |
| D0CBY0 | Putative signal peptide peptidase SppA, 36K type | 1.579574121 | 0.00154543 |
| D0CE32 | Curli production assembly/transport component CsgG | 0.378417893 | 7.66E-05 |
| D0CC21 | TonB-dependent siderophore receptor | 0.515071706 | 0.000643052 |
| D0CBQ4 | GTPase Era | 1.691283901 | 0.004878392 |
| D0C8N6 | OmpA family protein | 0.533941973 | 0.005362247 |
| D0CBK4 | Uncharacterized protein | 0.655233809 | 0.004613375 |
| D0C725 | Uncharacterized protein | 1.642542153 | 0.045990031 |
| D0C7A7 | Putative ATP synthase F0, A subunit | 0.504365089 | 0.001215292 |
| D0CCK7 | Carbonate dehydratase (EC 4.2.1.1) | 0.473044493 | 0.002141593 |
| D0CBP8 | Periplasmic serine endoprotease DegP-like (EC 3.4.21.107) | 0.586896897 | 0.000540661 |
| D0CBL3 | Tol-Pal system protein TolB | 0.639737934 | 0.001238531 |
| D0C805 | Glutamate/aspartate transport system permease protein GltK | 0.619377261 | 0.004236353 |

**Supplementary Table 9.** The list of significantly differentially expressed peptides under polymyxin B pressure (filtered by p˂0.05, fold change˃1.5) obtained by targeted proteomics.

| **Peptides** | **Protein name** | **Fold.Change** | **p value** |
| --- | --- | --- | --- |
| APDDEIALQHAR | D0C8K4 | 0.278833564 | 0.00066591 |
| AEFFDPSLDK | D0C8K4 | 0.257930499 | 0.000597742 |
| TSEVVHSVQGAGEYALGAR | D0CE32 | 0.126689648 | 0.000664192 |
| EVLGFGTTASYDSTLNGK | D0CE32 | 0.173545624 | 0.00068156 |
| VLDLAVR | D0CE32 | 0.161254644 | 0.000398507 |
| EAVNNLVTDIQNNR | D0CE32 | 0.128694661 | 0.000653075 |
| VITPDLK | D0C8N6 | 0.595433711 | 0.022112297 |
| IIEFESGSAVLTEAGQK | D0C8N6 | 0.549449723 | 0.030863347 |
| AEQDDTYAGGQVATSSNVGFLGSK | D0CC21 | 0.479913896 | 0.029382838 |
| TLLDVGAR | D0CC21 | 0.434356821 | 0.007419574 |
| LDAIAPGTYENLR | D0C7T2 | 0.602221393 | 0.004880661 |
| IAEDLAINR | D0C7T2 | 0.538877452 | 0.021075982 |
| ITPLQDGQTTDLISVR | D0C7T2 | 0.640716326 | 0.04266357 |
| HILIQPSEVVSPENAK | D0C7T2 | 0.515254002 | 0.032811196 |
| QIIDSIYK | D0C7T2 | 0.571814787 | 0.039468766 |

**Supplementary Table 1~7.** Shown as excel format.
